# Melatonin prevents chronic intermittent hypoxia-induced injury by inducing sirtuin 1-mediated autophagy in steatotic liver of mice

**DOI:** 10.1007/s11325-018-1741-4

**Published:** 2018-11-08

**Authors:** Jie Ren, Meng Jin, Zhen-xi You, Miao Luo, Yin Han, Guang-cai Li, Hui-guo Liu

**Affiliations:** Department of Respiratory and Critical Care Medicine, Tongji Hospital, Huazhong University of Science and Technology, Wuhan, 430030 Hubei China

**Keywords:** Melatonin, Liver, Chronic intermittent hypoxia, Sirtuin 1, Autophagy

## Abstract

**Background:**

Hepatic steatosis that occasionally results in nonalcoholic steatohepatitis (NASH) is related to obstructive sleep apnea (OSA). Many studies have shown that autophagy exerts protective effects on liver damage caused by various diseases and melatonin exhibits hepatoprotective properties. However, the mechanisms of liver injury induced by chronic intermittent hypoxia (CIH) and the effect of melatonin on the regulation of liver injury remain unclear.

**Purpose:**

This study was aimed to evaluate the role of CIH in steatohepatitis progression and the regulatory function of melatonin on fatty liver sensitivity to CIH injury, mainly focusing on autophagy signaling.

**Methods:**

A high-fat diet (FD)-induced obesity mouse model was subjected to intermittent hypoxia/normoxia events for approximately 8 h per day using an autophagy agonist, rapamycin, or an inhibitor, 3-methyladenine (3-MA), and SRT1720, a sirtuin 1 (SIRT1) activator, or sirtinol, a SIRT1 inhibitor, with or without melatonin for a total of six successive weeks, followed by assessment of expression of autophagy-related genes and activity of serum aminotransferase as well as histological evaluation of tissue morphology.

**Results:**

Neither FD nor CIH alone causes significant liver injury; however, the combination yielded higher serum aminotransferase activities and more severe histological changes, accompanied by a decrease in autophagy activity. Melatonin markedly inhibited FD/CIH-stimulated liver injury by enhancing autophagy. In contrast, SIRT1 inhibition resulted in a decrease in the expression of melatonin-induced autophagy-related genes as well as diminished its protective effects on FD/CIH-induced liver injury.

**Conclusion:**

These results suggest that melatonin could ameliorate FD/CIH-induced hepatocellular damage by activating SIRT1-mediated autophagy signaling.

## Introduction

Obstructive sleep apnea (OSA) pertains to the repeated upper airway collapse during sleep, resulting in chronic intermittent hypoxia (CIH). OSA is a commonly occurring disease that develops in 4–10% of adults [1]. However, among obese individuals, its prevalence is higher (40–60%) [2]. Obesity is also recognized as a risk factor for the development of liver steatosis [3, 4], which is the initial stage in the pathogenesis of steatohepatitis [5]. Increasing evidence indicates the association of OSA with non-alcoholic steatosis hepatitis (NASH), as well as chronic liver injury among obese individuals [6]. In addition, OSA has also been linked to visceral fat accumulation [7], which increases the risk for other obesity-related disorders. OSA has also been directly associated with higher risk for non-alcoholic fatty liver disease (NAFLD) and NASH, as well as fibrosis [8]. Human studies [7, 9] have shown that OSA, independent of obesity factors, leads to liver damage and NAFLD, and CIH plays a key role in liver injury [10]. Therefore, CIH may promote the progression from non-alcoholic fatty liver (NAFL) to NASH. However, its underlying mechanism remains elusive.

Autophagy pertains to intracellular catabolic recycling, allowing the delivery of cytoplasmic materials to lysosomes. Autophagy was initially described as a non-selective degradative mechanism. However, under specific conditions, which include NAFL, autophagy can act as a defense mechanism against NASH by selectively degrading various cytoplasmic lipid droplets as well as damaged organelles [11, 12]. Thus, autophagy plays a major role in the maintenance of lipid homeostasis in various organisms, as well as preventing steatosis progression [13].

Sirtuin 1 (SIRT1), which is a NAD+-dependent class III protein deacetylase, controls lipid metabolism [14] and inflammatory responses [15] in the liver. Previous studies have indicated that SIRT1 is a major regulator of autophagy [16, 17]. In addition, in hepatic ischemia/reperfusion, the pharmacologic stimulation of SIRT1 results in autophagy and enhanced liver function [18].

Melatonin (*N*-acetyl-5-methoxytryptamine) is an indoleamine that is mainly synthesized in the pineal gland of mammals and humans [19, 20] and has been shown to possess anti-oxidant [21], anti-inflammatory [22], and anti-apoptotic [23], as well as anti-autophagic [24] activities. Increasing evidence shows that melatonin imparts beneficial effects on hepatic injuries and diseases, which include ischemia/reperfusion [25], hepatocarcinoma [26], NAFL [27], viral hepatitis [28], and liver fibrosis [29]. However, animal investigations on the influence of melatonin on CIH-induced liver injury in vivo are limited.

The present study explored the protective activity of melatonin on CIH-induced hepatocellular damage using a high-fat-diet mouse model, mainly focusing on autophagic signaling.

## Materials and methods

### Animals

C57BL/6 male mice (weight range, 19–21 g) were obtained from Shanghai Silake Ltd., Inc. (Shanghai, China). The animals were maintained in a temperature- and humidity-controlled room (25 ± 1 °C and 55 ± 5%, respectively) under a 12-h light-dark cycle and fed a high-fat diet (FD) or a control diet (CD).

Induction of hepatocellular damage using CIH was performed as previously reported [10]. The mice were kept in special cages that were equipped with a controlled gas delivery system that provided regulated air, nitrogen, and oxygen flow. The fraction of inspired oxygen (FiO_2_) in the cage of the CIH group was decreased from 21 to 5–6% for 15–20 s in 1 min, and then immediately increased to 21% using rapid oxygenation to room air levels in the following 1-min period. Gas-flow exposure of the normoxia groups was similar to that of the CIH groups, except that only room air was employed. The CIH groups were exposed to intermittent hypoxia for approximately 8 h per day for a total of six weeks.

During CIH, melatonin (Sigma Chemical Co., St. Louis, MO, USA) dissolved in 5% ethanol in saline was administered intraperitoneally (10 mg/kg/day) for a total of six successive weeks. In addition, sirtinol (Abcam, Cambridge, MA, USA) dissolved in 2% DMSO-saline was administered intraperitoneally (10 mg/kg/day) for a total of six successive weeks during CIH. SRT1720 (20 mg/kg/day body weight; AdooQ Bioscience, Irvine, CA, USA) was administered intraperitoneally for a total of six successive weeks during CIH. 3-Methyladenine (3-MA) (Sigma-Aldrich, St. Louis, MO, USA) was administered intraperitoneally (30 mg/kg/day) for a total of six successive weeks during CIH. Rapamycin (Sigma-Aldrich, St. Louis, MO, USA) was administered intraperitoneally (1 mg/kg/day) for a total of six successive weeks during CIH.

### Biochemical determinations

Serum alanine (ALT), aspartate (AST) aminotransferase, and lactate dehydrogenase (LDH) activities were evaluated at 37 °C by assessing for a decrease in absorbance at a wavelength of 340 nm for 1 min, which is caused by the disappearance of NADH, with ChemiLab ALT, AST, and LDH assay kits (IVD Lab Co., Ltd., Uiwang, Korea), respectively, and a Hitachi 7600 automatic analyzer (Tokyo, Japan).

### Histological assay

Within 24 h of the last exposure, the mice were euthanized via intraperitoneal injection of 10% chloral hydrate (0.3 mL/100 g). Their livers were collected and then fixed in 10% buffered formalin solution, dehydrated across an ethanol gradient, and then embedded in paraffin for sectioning. The tissue sections (6-μm thick) were stained with hematoxylin and eosin (HE) (Jiancheng, Nanjing, China) as well as Masson’s trichrome. Histological assessment was conducted using an Olympus BX50 light microscope (Tokyo, Japan) and then evaluated by a single-blinded pathologist. The frozen sections were stained with HE according to standard protocol. The collagen fibers were stained blue using Masson’s trichrome and assessed for liver fibrosis. A terminal deoxynucleotidyl transferase-mediated dUTP nick-end labeling (TUNEL) kit (Roche, Germany) was employed to assess the degree of hepatic cell apoptosis. Histological alterations in randomly selected histological fields at a × 200 magnification were assessed. For quantification of histological alterations, the stained sections were graded according to Suzuki et al. [30]. Briefly, three liver injury indices were employed: sinusoidal congestion (score, 0–4), hepatocyte necrosis (score, 0–4), and ballooning degeneration (score, 0–4), with a total score of 0–12. Tissues without signs of congestion, necrosis, or ballooning were given a score of 0, whereas severe congestion/ballooning with > 60% lobular necrosis were given a score of 12.

### Transmission electron microscopy

Liver tissues were fixed in 2.5% glutaraldehyde and 4% paraformaldehyde dissolved in 100 mM sodium phosphate (pH 7.2). The tissues were washed using 100 mM Na cacodylate (pH 7.4), fixed in 2% osmium tetroxide, and then again washed. The fixed tissues were dehydrated across an ethanol gradient and propylene oxide and then embedded in epoxy resin (Taab 812 Resin; Canemco Inc., Montreal, QC, Canada). The resulting ultrathin (60–70 nm) sections were counterstained with uranyl acetate and lead citrate, and then viewed on a Hitachi 7600 transmission electron microscope (Hitachi High-Technologies America, Inc., Schaumburg, IL, USA) that was equipped with a MacroFire monochrome progressive scan CCD camera (Optronics, 10 Inc., Muskogee, OK, USA) with an AMTv image capture software (Advanced Microscopy Techniques, Inc., Danvers, MA, USA).

### Real-time quantitative RT-PCR

Liver tissues were employed for RT-PCR analysis. Total RNA was extracted with TRIzol (Life Technologies, Rockville, MD, USA) following the manufacturer’s instructions. Total RNA concentrations were determined by spectrophotometry, and RNA purity was evaluated based on the OD260/OD280 ratio. Total RNA was used for cDNA synthesis, and PCR was performed with SYBR Green. Gene-specific primers were developed using Premier 5.0 and sent to Sangon Biological Engineering Co., Ltd. (Shanghai, China) for synthesis. The following primers were used: *GAPDH* 5′-TGA AGG GTG GAG CCA AAA G-3′ and 5′-AGT CTT CTG GGT GGC AGT GAT-3′; *Beclin-1* 5′-GGA ATG AAA TCA ATG CTG CCT-3′ and 5′-CCC CAG AAC AGT ATA ACG GCA-3′; *Atg12* 5′-CAT CCT GCT GAA GGC TGT AGG-3′ and 5′-AAC AAC TGT TCC GAG GCC AC-3′; *Atg5* 5′-GCC ATC AAC CGG AAA CTC AT-3′ and 5′- TCC AGC ATT GGC TCT ATC CC-3′; *Atg3* 5′-GAA GGG AAA GGC TCT GGA AGT-3′ and 5′- TTG CCA TGT TGG ACA GTG GT-3′; *Atg7* 5′-TGG GAG AAG AAC CAG AAA GGA-3′ and 5′- CAG GCA CTT GAC AGA CAC GAC-3′; *p62* 5′-AGT GGA CCC ATC TAC AGA GGC-3′ and 5′- GGT CTG TAG GAG CCT GGT GAG-3′; and *SIRT1* 5′-CGG TAT CTA TGC TCG CCT TG-3′ and 5′-ACA GAG ACG GCT GGA ACT GTC-3′. The PCR conditions were as follows: pre-denaturation at 95 °C for 30 s; and then 40 cycles of 1 min at 93 °C, 1 min at 55 °C, and 1 min at 72 °C. For every gene, “no-template” and “no-amplification” controls were employed. Then, melting curve analysis was conducted to assess the specificity of the PCR products based on their specific melting temperatures as follows: denaturation at 95 °C for 15 s, followed by a decrease in temperature to 60 °C for 1 min, and then an increase to 95 °C for 15 s. Data normalization was performed using GAPDH. mRNA expression levels of Beclin-1, Atg12, Atg5, Atg3, Atg7, p62, and SIRT1 were presented based on their changes compared to the control group.

### Western blot analysis

Total protein was isolated by homogenizing each liver tissue sample of every mouse in PRO-PREPTM Protein Extraction Solution (iNtRON Biotechnology Inc., Seongnam, Korea). Protein concentrations were measured using a BCA Protein Assay kit (Pierce Biotechnology Inc., Rockford, IL, USA). Protein samples (16 μg) from every sample were resolved in a 10% SDS-PAGE gel and then immunoblotted onto a 0.45-μm Immobilon-P polyvinylidene difluoride membrane (Bio-Rad Laboratories, Hercules, CA, USA). The membranes were blocked with 5% skim milk powder in Tris-buffered saline with Tween 20 (TBST) and then incubated overnight at 4 °C with the primary antibodies. Then, the primary antibodies were washed with TBST five times for 7 min each time, incubated with the corresponding secondary antibodies, and then detected with an ECL detection system (iNtRON Biotechnology Inc., Seongnam, Korea). The intensity of the protein reaction bands was evaluated using an ECL western blotting detection system. The primary antibodies employed were as follows: Atg3, Atg12-5 complex, Atg7, and SIRT1 (Cell Signaling Technology, Beverly, MA, USA); p62, Beclin-1, and LC3 (Abcam, Cambridge, MA, USA); and GAPDH (Sigma-Aldrich, St. Louis, MO, USA). Protein densities were normalized to that of GAPDH. Western blotting was performed for a least three times.

### Data analysis

The data were presented as the means ± standard deviation (SD) and then compared using one-way ANOVA and the Student-Newman-Keuls test. Significant differences were defined as those with a *P* value < 0.05. Statistical analysis was conducted using GraphPad Prism 5 (La Jolla, CA, USA).

## Results

### CIH results in liver injury in FD mice

**Fig. 1:**
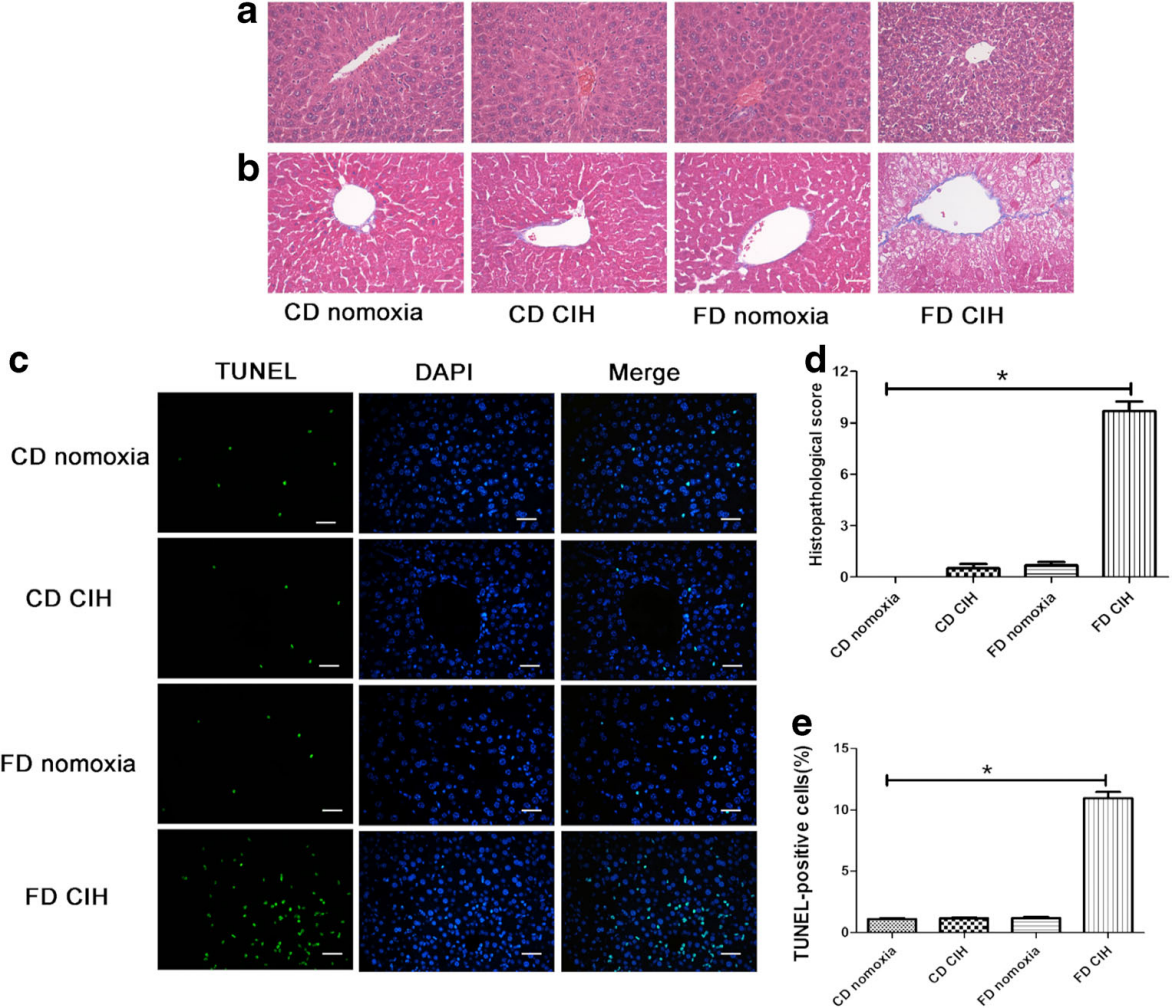
Histopathological alterations involving liver tissues. (A) Representative images of HE-stained liver tissues of the four groups (original magnification, × 200). (B) Hepatic fibrosis in the four groups as detected by Masson’s trichrome (original magnification, × 200). (C) Representative images of TUNEL-stained liver tissues of the four study groups (original magnification, × 200). Scale bar = 20 μm. (D) Extent of liver injury in the four groups graded using the Suzuki score. (E) Percentage of TUNEL-positive cells among the four groups. *N* = 3, all data are presented as the mean ± SD. *P < 0.05

The degree of hepatocyte injury was evaluated based on serum ALT and AST levels as well as histopathological alterations of the liver tissues. Histopathological assessment indicated that the CD normoxia group had normal hepatic lobular architecture and cellular morphology (Suzuki score, 0.0 ± 0.0). However, the CD/CIH and FD normoxia groups showed only slight fatty degeneration in the liver tissues, which mainly involved zone I hepatocytes (Suzuki score, 0.7 ± 0.4 and 0.9 ± 0.3, respectively; all *P* > 0.05). Numerous areas of steatosis, extensive necrosis and intracellular vacuolization, and moderate production of inflammatory exudates were detected in the FD/CIH group (9.7 ± 0.8; *P* < 0.05) (Fig. 1(A, D)). Masson’s trichrome staining revealed fibrosis of the hepatic tissue. No differences in fibrosis levels involving the hepatic tissues were observed among the CD normoxia, CD/CIH, and FD normoxia groups (*P* > 0.05). However, more severe fibrosis of the liver portal and periportal areas were observed in the FD/CIH group relative to the other three groups (*P* < 0.05), with perisinusoidal fibrosis (Fig. 1(B)). No significant difference in the rate of TUNEL-positive cells was observed among the CD normoxia (1.08 ± 0.07%), CD/CIH (1.14 ± 0.05%), and FD normoxia (1.25 ± 0.33%) groups (*P* > 0.05), but was relatively lower than that of the FD/CIH (10.96 ± 0.8%) group (*P* < 0.05) (Fig. 1(C, E)).

Serum ALT, AST, and LDH levels are major indicators of liver injury. Table 1 shows that the serum ALT and AST activities of the FD normoxia group were significantly higher relative to those of the CD normoxia group. The serum ALT and AST activities of the FD/CIH group respectively increased to 169.49 ± 8.76 U/L and 303.91 ± 13.68 U/L, which were significantly higher compared to the CD normoxia and FD normoxia groups (all *P* < 0.05). No significant difference in LDH levels was observed among the CD normoxia, CD/CIH, and FD normoxia groups (*P* > 0.05). However, the serum LDH activity of the FD/CIH group significantly increased to 2242.42 ± 192.82, which was markedly higher than that of the CD normoxia group (all *P* < 0.05).

**Table 1:** The serum levels of biochemical changes

	CD normoxia	CD CIH	FD normoxia	FD CIH
Serum ALT (U/L)	50.82 ± 7.25	58.05 ± 8.08	91.38 ± 7.73*	169.49 ± 8.76*^#^
Serum AST (U/L)	78.24 ± 15.48	85.13 ± 9.40	138.20 ± 13.90*	303.91 ± 13.68*^#^
Serum LDH (U/L)	958.15 ± 111.66	968.44 ± 75.48	995.91 ± 81.83	2242.42 ± 192.82*

Values are expressed as means ± SD

*N* = 5, *p < 0.05 versus CD normoxia; ^#^*p* < 0.05 versus FD normoxia

### CIH leads to decreased autophagy in steatotic hepatocytes

**Fig. 2:**
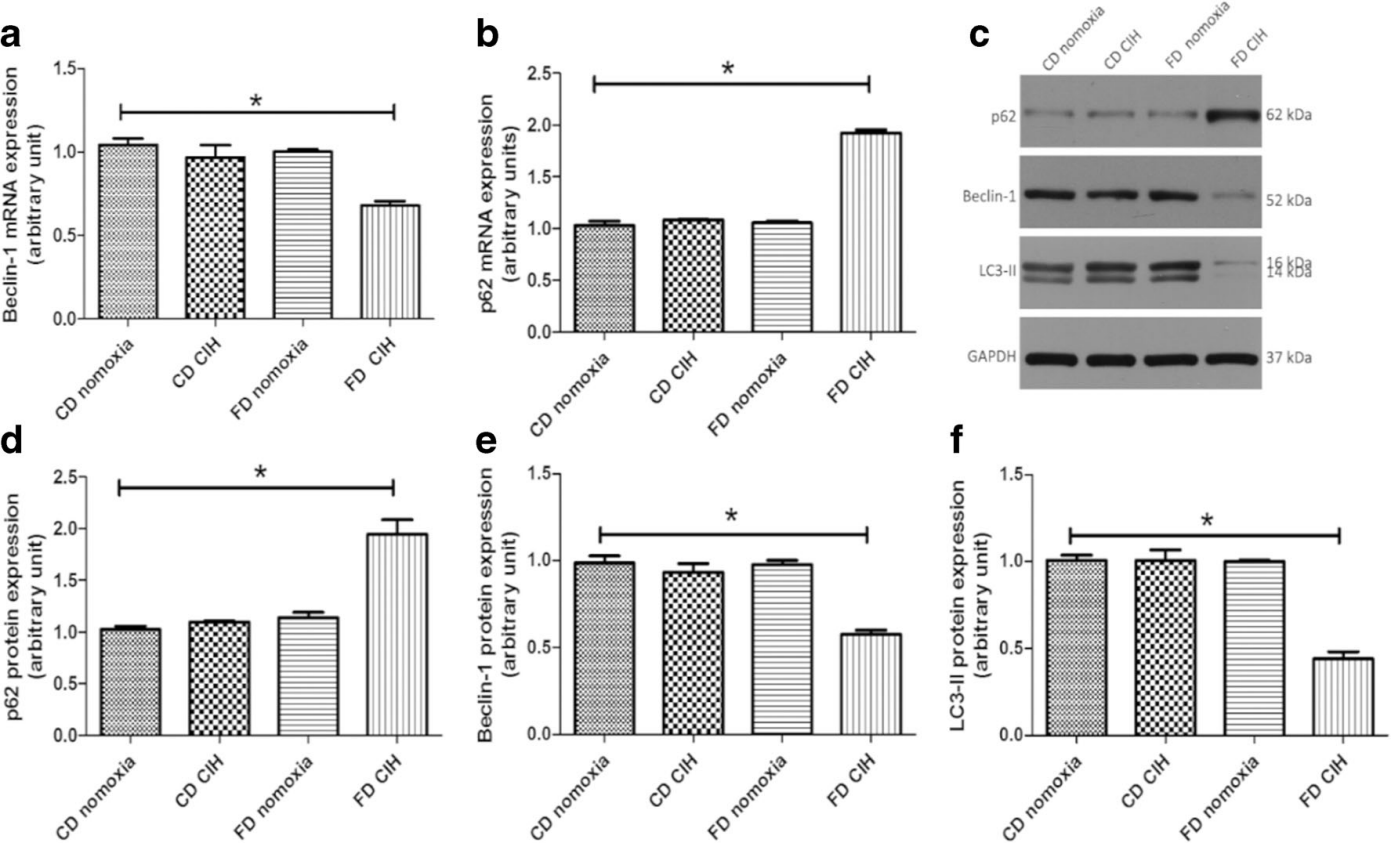
CIH decreases autophagic flux in steatotic hepatocytes. **a** Beclin-1 mRNA expression in the four groups. **b** p62 mRNA expression in the four groups. **c** p62, LC3-II, and Beclin-1 protein expression. **d**–**f** Densitometric evaluation of western blotting results. *N* = 3, all data are presented as the mean ± SD. *P < 0.05

To investigate the influence of FD/CIH on hepatocellular autophagy, we assessed the expression of autophagy markers LC3-II, Beclin-1, and p62 using western blotting or PCR. LC3-II is a molecular marker of autophagosomes and plays a crucial role in the establishment of the structure and function of autophagosomes [31]. The CD/CIH and FD normoxia groups showed a decrease in LC3-II and Beclin-1 expression levels relative to the CD normoxia group; however, the differences were not statistically significant (*P* > 0.05). In addition, the FD/CIH group exhibited a significant decrease in the levels of LC3-II protein expression and Beclin-1 mRNA and protein expression to around 43.14%, 67.90%, and 51.25% of the CD normoxia group (Fig. 2a, c, e, f; *P* < 0.05). SQSTM1/p62, which is a cargo adaptor for autophagy, is a marker for autophagic flux [32]. CIH did not induce changes in the expression levels of p62 mRNA and protein in the CD group (*P* > 0.05), and no difference between the FD normoxia and CD normoxia groups was observed. However, the FD/CIH group exhibited a significant increase in p62 mRNA and protein expression levels, which were respectively 1.95-fold and 1.8-fold higher than the CD normoxia group (*P* < 0.05) (Fig. 2b–d). In summary, these results indicate an inhibition of autophagy in the mouse FD/CIH model.

### Enhanced autophagy ameliorates FD/CIH-induced liver injury

**Fig. 3:**
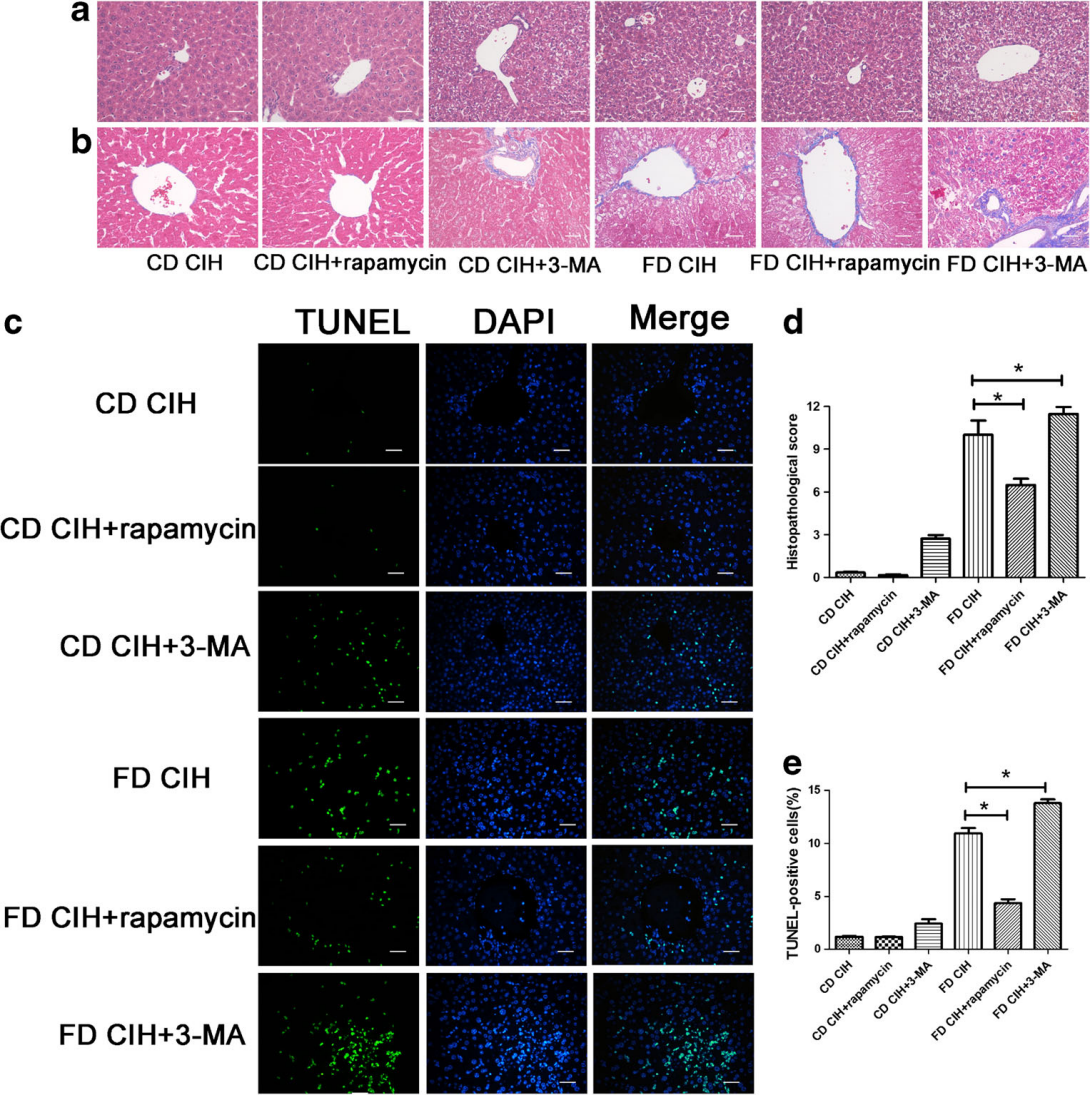
Histological changes induced by autophagy activation or inhibition. (A) Representative images of HE-stained liver tissues in six different groups (original magnification, × 200). (B) Hepatic fibrosis in the six groups as detected by Masson’s trichrome (original magnification, × 200). (C) Representative images of TUNEL-stained liver tissues from the six groups (original magnification, × 200). Scale bar = 20 μm. (D) Extent of liver injury in the six groups as graded using the Suzuki score. (E) Percentage of TUNEL-positive cells in six different groups. *N* = 3, the data are presented as the mean ± SD. *P < 0.05

Then, we investigated the role of autophagy in FD/CIH-induced liver injury. Treatment with rapamycin, an agonist of autophagy, for six successive weeks protected mice against liver injury; in contrast, treatment with 3-MA, an inhibitor of autophagy, for six successive weeks further aggravated liver injury. Compared to the FD/CIH group, the structure and histology of the liver after rapamycin treatment were well preserved, including decreased necrosis and less steatosis or fibrosis. Thus, treatment with rapamycin significantly ameliorates liver damage. In contrast, the autophagy inhibitor 3-MA (5 mM) induced further aggravation of liver damage relative to the FD/CIH group (*P* < 0.05) (Fig. 3(A, B, D)); Moreover, using the TUNEL assay, treatment with rapamycin was shown to significantly decrease hepatocyte apoptosis, whereas 3-MA increased it (*P* < 0.05) (Fig. 3(C, E)).

### SIRT1 activation attenuates FD/CIH-induced liver injury

**Fig. 4:**
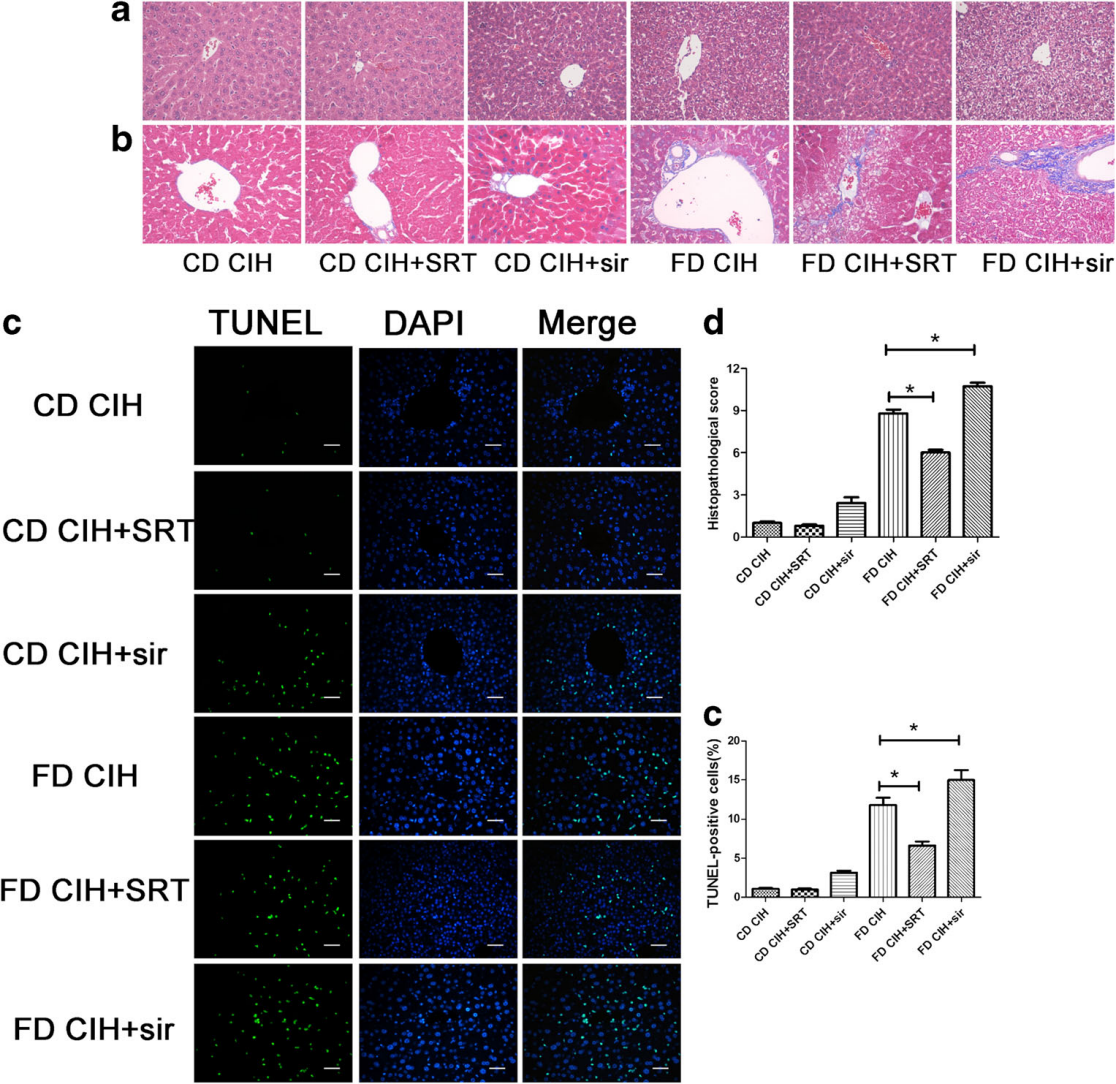
Histological changes induced by SIRT1 activation or inhibition. (A) Representative images of HE-stained hepatic tissues of the four groups (original magnification, × 200). (B) Hepatic fibrosis in the four groups as detected by Masson’s trichrome (original magnification, × 200). (C) Representative images of TUNEL-stained in liver tissues of the four groups (original magnification, × 200). Scale bar = 20 μm. (D) Extent of liver injury in the four groups as graded using the Suzuki score. (E) Percentage of TUNEL-positive cells in the four groups. *N* = 3, the data are presented as the mean ± SD. *P < 0.05; SRT, SRT1720; sir, sirtinol

Histologic assessment of the livers of the FD/CIH group by HE and Masson’s trichrome staining revealed significant areas of necrosis and congestion extending toward the portal triad. This was markedly improved with treatment using SRT1720, a selective SIRT1 activator, where necrosis was limited to the immediate centrilobular area. However, liver damage was more severe when SIRT1 was inhibited with sirtinol (Fig. 4(A, B)). The extent of hepatic injury was semiquantitatively represented using the Suzuki scoring system (Fig. 4(D)). Moreover, using a TUNEL assay, pretreatment with SRT1720 was shown to significantly decrease hepatocyte apoptosis compared to that in the FD/CIH group (*P* < 0.05). Conversely, SIRT1 inhibition by sirtinol treatment further increased necrosis and apoptosis compared to that in mice treated with FD/CIH (*P* < 0.05) (Fig. 4(C, E)).

### Melatonin ameliorates FD/CIH-induced hepatocellular damage

**Fig. 5:**
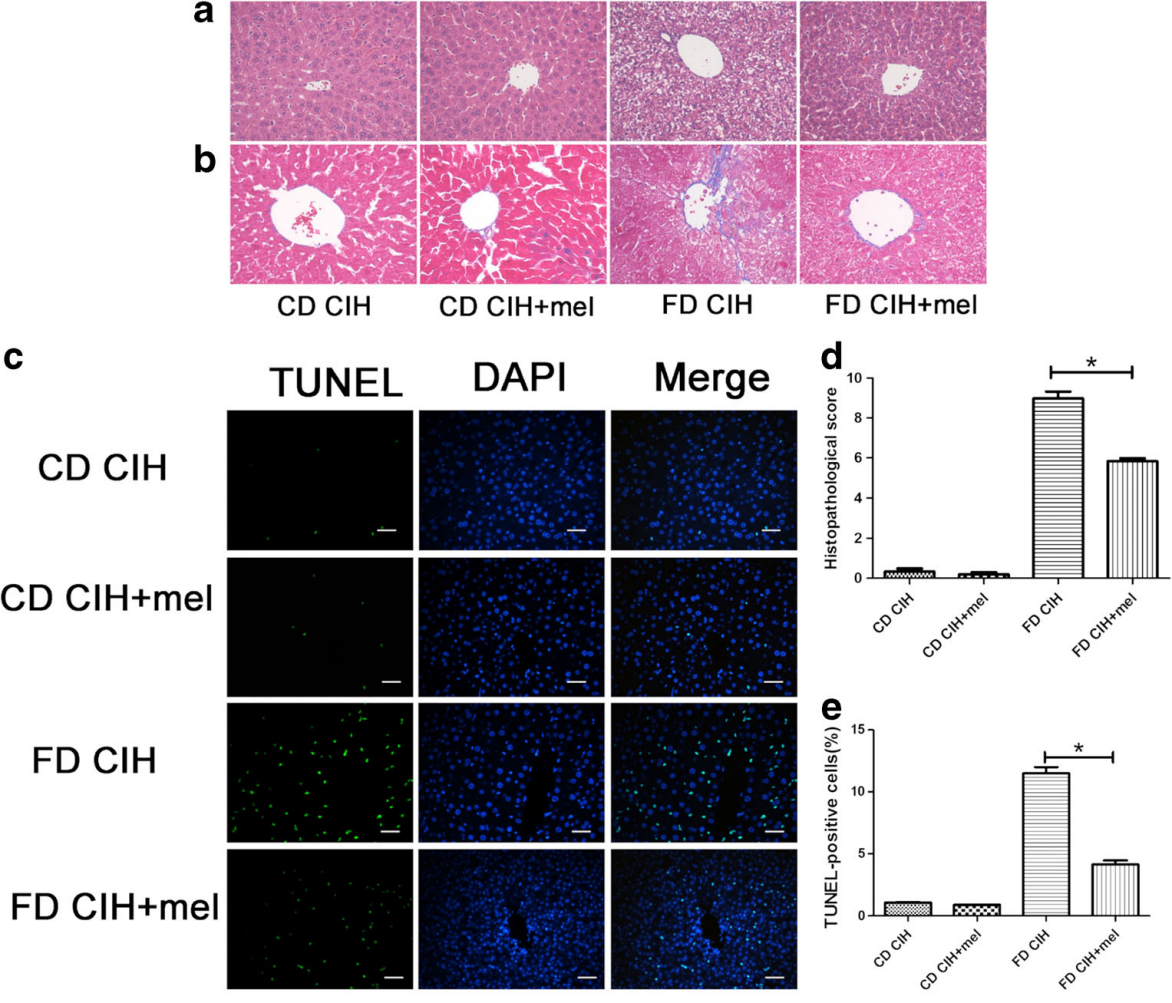
Effect of melatonin on FD/CIH-induced histological changes involving the liver. (A) Representative images of HE-stained hepatic tissues of the four groups (original magnification, × 200). (B) Hepatic fibrosis in the four groups as detected by Masson’s trichrome (original magnification, × 200). (C) Representative images of TUNEL-stained liver tissue of four study groups (original magnification, × 200). Scale bar = 20 μm. (D) Extent of liver injury in the four groups as graded using the Suzuki score. (E) Percentage of TUNEL-positive cells among the four groups. *N* = 3, the data are presented as the mean ± SD. *P < 0.05; mel, melatonin

The effect of melatonin on FD/CIH-induced liver injury was histologically assessed. The CD/CIH group exhibited slight, interspersed fatty degeneration of hepatic lobules (0.2 ± 0.0). Histopathological assessment of the melatonin-treated (CD/CIH+mel) group indicated normal hepatic lobular organization and cellular morphology (Suzuki score, 0.0 ± 0.0), which was similar (*P* > 0.05) to that observed in the CD/CIH group. Melatonin administration (5.7 ± 0.2) ameliorated FD/CIH-induced liver damage (9.2 ± 0.6) (*P* < 0.05) (Fig. 5(A, B, D)). The percentage of TUNEL-positive cells was also reduced after treatment with melatonin (Fig. 5(C, E); FD/CIH group, 11.96 ± 0.35%; FD/CIH+mel, 4.96 ± 0.40%).

### Melatonin restores impaired autophagy in FD/CIH-induced liver injury

**Fig. 6:**
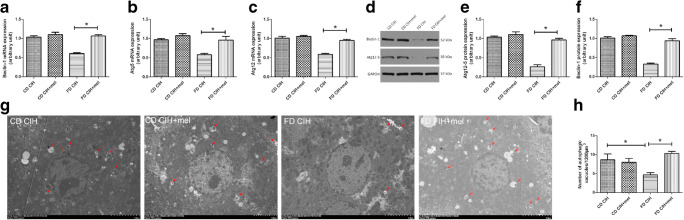
Melatonin reverses impairment of autophagy. **a**–**c** mRNA expression levels of Beclin-1 (**a**), Atg5 (**b**), and Atg12 (**c**) among the four different groups. **d** Protein expression levels of Atg12-5 and Beclin-1. **e**, **f** Densitometric assessment of western blotting results. **g**, **h** Representative TEM images of hepatic tissues among four different groups, arrows show autophagosomes. Scale bar = 5 μm, *N* = 3, the data are presented as the mean ± SD. *P < 0.05; mel, melatonin

Figure 6 shows that the mRNA or protein expression levels of autophagy-related genes of the CD/CIH and CD/CIH+mel groups did not significantly differ (*P* > 0.05). The FD/CIH group showed lower Atg12-5 and Beclin-1 expression levels compared to the CD/CIH and CD/CIH+mel groups (Fig. 6a–f). Melatonin treatment resulted in a significant increase in Atg12-5 and Beclin-1 protein and mRNA expression levels relative to the FD/CIH group (*P* < 0.05).

To further demonstrate the effects of melatonin treatment on hepatic autophagy, we performed TEM, which is the gold standard for autophagy. The FD/CIH group exhibited a significant decrease in the number of autophagosomes compared to the CD normoxia group, which was reversed by the application of melatonin (Fig. 6g, h).

### Melatonin increases autophagosome formation in FD/CIH-induced liver injury

**Fig. 7:**
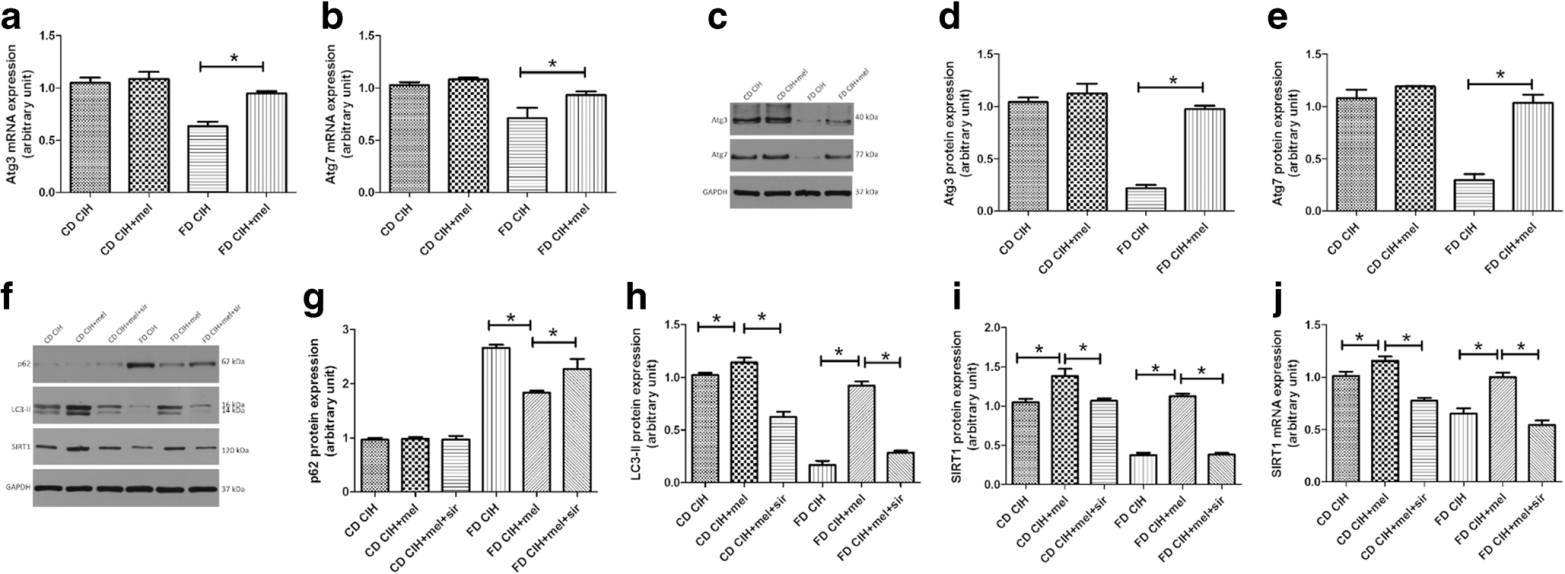
Melatonin reverses impairment of autophagy and triggers sirtuin 1 signaling. Atg3 (**a**) and Atg7 (**b**) mRNA expression in the four groups. **c** Atg3 and Atg7 protein expression. **d**, **e** Quantification of Atg3 and Atg7 protein expression in the four groups. **f** p62, LC3-II, and SIRT1 protein expression. **g**, **h**, **i** Assessment of p62, LC3-II, and SIRT1 protein expression levels in the four groups. **j** SIRT1 mRNA expression in the four groups. *N* = 3, the data are presented as the mean ± SD. *P < 0.05; mel, melatonin; sir, sirtinol

Next, we detected the expression levels of key autophagy-related proteins, Atg3 and Atg7, which could impair autophagosome formation when knocked out [33]. Figure 7a–e show a reduction in the Atg3 and Atg7 mRNA and protein levels of expression in the FD/CIH group relative to the CD/CIH and CD/CIH+mel groups (*P* < 0.05). The application of melatonin resulted in an increase in expression levels (*P* < 0.05), although these were still lower compared to that of the CD/CIH and CD/CIH+mel groups. No statistically significant differences were observed between the CD/CIH and CD/CIH+mel groups (*P* > 0.05).

### Melatonin activates SIRT1 signaling in FD/CIH-induced liver injury

To determine whether melatonin-induced activation of autophagy during liver injury in FD/CIH is mediated by SIRT1, we assessed the mRNA and protein levels of SIRT1. The FD/CIH group showed a significant decrease in SIRT1 mRNA and protein expression levels to respectively 74.5% and 46.1% relative to the CD/CIH group, and this effect was reduced by melatonin treatment. In addition, compared to the FD/CIH+mel group, SIRT1 inhibitor, sirtinol, pretreatment effectively inhibited SIRT1 mRNA and protein expression (42.5% and 51.8% of those observed in the FD/CIH+mel group, respectively) (Fig. 7f, i, j). Furthermore, melatonin-induced LC3-II upregulation and of p62 downregulation were effectively abolished by the application of sirtinol (23.8% and 1.3-fold of that observed in the FD/CIH+mel group, respectively) (Fig. 7f–h). Sirtinol treatment resulted in the reversal of the protective effect of melatonin from FD/CIH-induced liver injury, as shown by significantly elevated serum ALT levels (FD/CIH+mel, 82.28 ± 9.84 U/L vs. FD/CIH+mel+sir, 149.64 ± 8.47 U/L), AST levels (FD/CIH+mel, 129.27 ± 12.77 U/L vs. FD/CIH+mel+sir, 268.48 ± 16.47 U/L), and LDH levels (FD/CIH+mel, 1658.10 ± 280.95 U/L vs. FD/CIH+mel+sir, 2083.40 ± 272.10 U/L) compared to the FD/CIH+mel group (Table 2).

**Table 2:** The serum levels of biochemical changes

	CD CIH	CD CIH+mel	CD CIH+mel+sir	FD CIH	FD CIH+mel	FD CIH+mel+sir
Serum ALT (U/L)	58.05 ± 8.08	57.59 ± 8.47	54.36 ± 7.47	169.49 ± 8.76	82.28 ± 9.84*	149.64 ± 8.47^#^
Serum AST (U/L)	85.13 ± 9.40	81.78 ± 9.46	88.37 ± 9.03	303.91 ± 13.68	129.27 ± 12.77*	268.48 ± 16.47^#^
Serum LDH (U/L)	1229.44 ± 75.48	1203.95 ± 87.16	1219.37 ± 85.74	2242.42 ± 192.82	1658.10 ± 280.95*	2083.40 ± 272.10^#^

Values are expressed as means ± SD

*N* = 5, *p < 0.05 versus FD CIH; ^#^*p* < 0.05 versus FD CIH+mel; mel, melatonin; sir, sirtinol

## Discussion

CIH is regarded as an independent factor that is involved in the pathogenesis of NAFLD [34], and OSA has been reported to induce the progression of steatosis into NASH [35]. The present study established a CIH murine model to elucidate the mechanism underlying CIH-induced hepatic injury. Our results support our hypothesis that (1) autophagy is inhibited during FD/CIH-induced liver injury; (2) the activation of SIRT1 induces an increase in autophagy; (3) melatonin decreases FD/CIH-induced liver injury; (4) melatonin imparts a protective effect on the liver via activation of autophagy; and (5) SIRT1 upregulation is involved in melatonin-induced autophagy and protection against liver injury.

A previous study has shown that liver dysfunction and histological damage are aggravated by high fat and intermittent hypoxia compared to high fat alone [36]. The assessment of 101 obese subjects revealed a positive correlation between CIH and the degree of liver injury [37]. Chin et al. found that 30% of obese patients experiencing sleep apnea as well as hypopnea syndrome had higher transaminase levels [38, 39]. Our findings indicate that the CIH of sleep apnea further aggravates the progression of simple fatty liver to NASH as well as hepatic fibrosis. In the present study, six weeks of high fat diet alone did not cause liver damage. However, CIH may exacerbate liver damage in the steatotic liver, as indicated by the extensive deterioration of hepatic function and histological alterations.

The pathogenesis of NASH is complex, and the most tenable mechanism involves the “two-hit theory” [40, 41]. Autophagy pertains to a lysosome-dependent mechanism wherein dysfunctional or damaged intracellular organelles are recycled using lysosomes. The first hit involves intracellular lipid deposition, particularly triglycerides, which is prerequisite for the pathogenesis of NAFLD. The second hit involves oxygen stress and lipid peroxidation, which are essential to NASH progression. CIH further increases oxidative stress by enhancing reactive oxidase activity and decreasing antioxidant enzyme activity [42]. Therefore, we have reason to believe that in this study, FD is the first hit, and CIH is the second hit, thereby resulting in NASH. Previous studies have shown that autophagy plays a major role in reperfusion injury [18, 43] and autophagy activation imparts a protective effect on liver injury in various liver injury models. It has also been suggested that autophagy may be involved in the NASH “two-hit theory” [44].

In the present study, we used a variety of experimental methods to test whether autophagy is involved in FD/CIH-induced liver damage. We initially examined changes in p62 protein expression. P62 is an autophagy-related gene that binds to LC3-II and functions as a cargo adaptor of autophagy [45]. An upregulation of p62 protein expression in the hepatic tissues of the FD/CIH group relative to that in the CD normoxia group was observed. The LC-3II protein is a marker of autophagy, and its expression level has been correlated with the number of autophagic vesicles [46]. We also detected a significant reduction in LC3-II and Beclin-1 expression in the hepatic tissues of the FD/CIH group relative to the CD normoxia group. The inhibition of autophagy under these conditions leads to an increase in hepatocyte apoptosis and more severe liver structural damage. In contrast, the induction of autophagy imparts a protective effect on animals with liver CIH injury. These findings provide evidence for our hypothesis that autophagy serves as a protective mechanism involving FD/CIH-induced liver injury, and autophagy activation may be a novel target for the treatment of CIH-induced liver injury.

Melatonin is considered to impart a strong protective effect on liver damage based on its antioxidant properties [25, 47]. Here, we employed a mouse model to investigate the protective effects of melatonin on FD/CIH-induced hepatocellular damage. The application of melatonin resulted in the amelioration of FD/CIH-induced hepatic injury by reducing the serum aminotransferase levels. Furthermore, the histopathologic changes in the liver also indicate the protective effect of melatonin. Evaluation of liver tissues of FD/CIH mice indicated numerous areas exhibiting steatosis and massive necrosis, which were reduced by the application of melatonin. These findings indicate that melatonin may be potentially employed as an effective therapeutic regimen against FD/CIH-induced liver injury.

Recent in vitro studies have shown that melatonin enhances autophagy, thereby imparting protective effects on mesenchymal stem cells [48] as well as placental nutrition [49]. However, whether the effect occurs in vivo remains unclear. Here, we show that FD/CIH leads to the upregulation of p62, which in turn is alleviated by melatonin. In addition, FD/CIH further decreases the expression of Atg12-5 conjugate, Atg3, Atg7, Beclin-1, and LC3-II, whereas melatonin attenuates these effects.

Several studies have shown that the inhibition of SIRT1 expression may result in the development of alcoholic as well as non-alcoholic fatty liver diseases [50, 51]. The present study has shown that inhibition of SIRT1 can lead to increased hepatocyte apoptosis and more severe liver structural damage than the FD/CIH group. Conversely, the upregulation of SIRT1 imparts a protective effect on liver FD/CIH injury. Several studies have revealed a positive correlation between SIRT1 expression and autophagy levels. In vitro studies have shown that the lack of SIRT1 may result in the methylation of autophagy-related genes, such as Atg5, Atg7, Atg8, and LC3 [52, 53]. In our study, FD/CIH induces a significant decrease in SIRT1 expression, and the application of melatonin reversed this process. This finding prompted us to further investigate whether SIRT1 contributes to the activation of autophagy by melatonin. Our findings indicate that SIRT1 inhibition eliminates the effect of melatonin on autophagy activation in the liver, which in turn confirms that melatonin-activated autophagy is SIRT1 dependent.

Taken together, our results indicate that CIH may be the “second hit” to a FD, which in turn, results in the pathogenesis of NASH by inhibiting autophagy. Furthermore, melatonin reverses the impairment of autophagy by regulating SIRT1 expression, thereby imparting a protective effect against FD/CIH-induced liver injury. Therefore, treatment with melatonin might be a useful pharmacological strategy to reduce NASH caused by obesity and CIH.
