# Correction to: Melatonin prevents chronic intermittent hypoxia-induced injury by inducing sirtuin 1-mediated autophagy in steatotic liver of mice

**DOI:** 10.1007/s11325-022-02656-5

**Published:** 2022-06-22

**Authors:** Jie Ren, Meng Jin, Zhen-xi You, Miao Luo, Yin Han, Guang-cai Li, Hui-guo Liu

**Affiliations:** Department of Respiratory and Critical Care Medicine, Tongji Hospital, Huazhong University of Science and Technology, Wuhan, 430030 Hubei China

**Correction to: Sleep and Breathing (2019) 23:825–836**

**https://doi.org/10.1007/s11325-018-1741-4**

In the article that appeared on Page: 825–836, Vol 23 (September 2019) of the *Sleep and Breathing* [1], one error was discovered in Fig. 4. Because of our carelessness in combining images, we put the wrong images on the CD CIH + SRT group in Fig. 4C, and the figure part of TUNEL-positive cells (%) in Fig. 4 should be denoted by e. The corrected version of Fig. 4 is presented here.

**Fig. 4:**
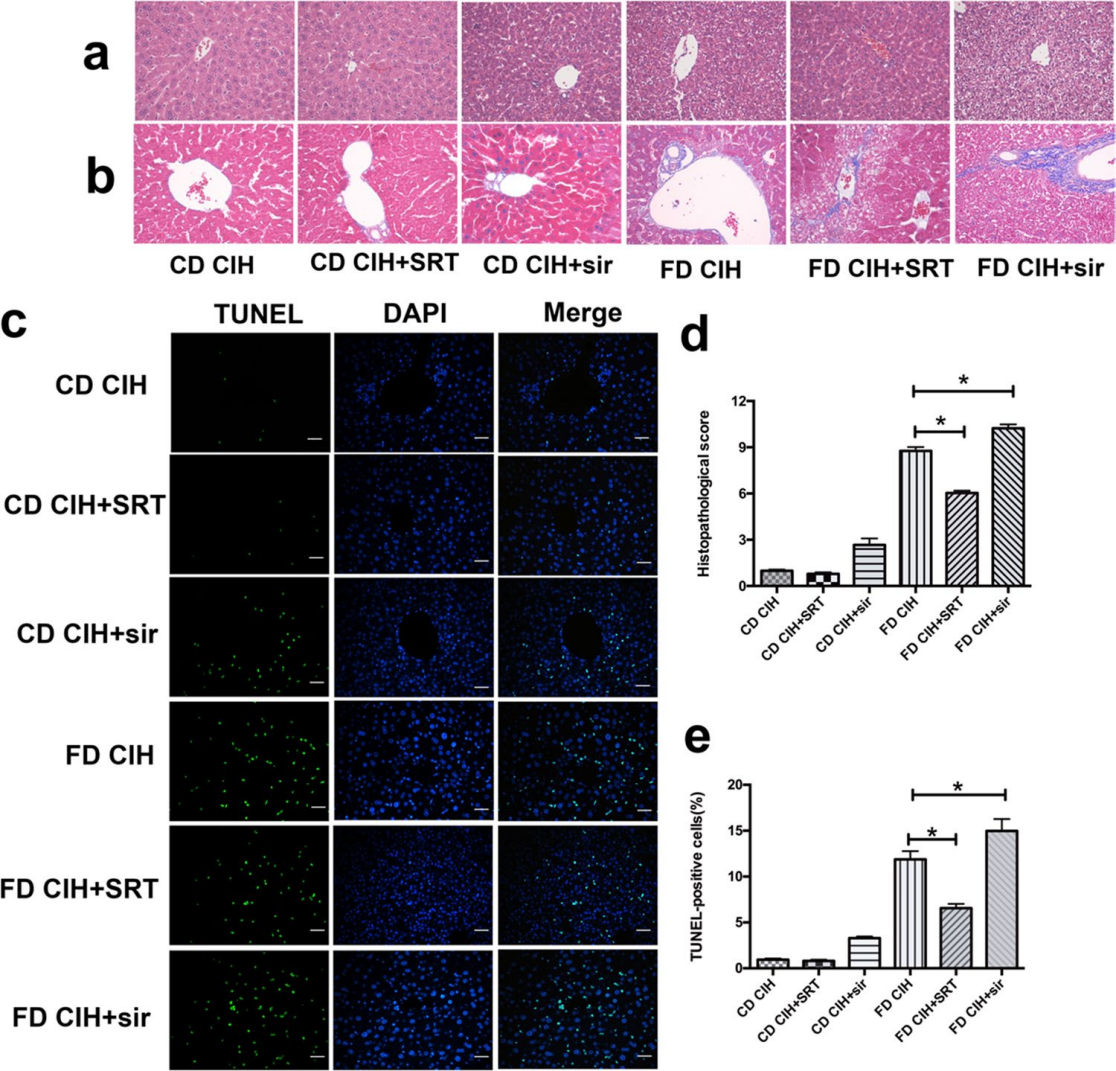
Histological changes induced by SIRT1 activation or inhibition. (**A**) Representative images of HE-stained hepatic tissues of the four groups (original magnification, × 200). (**B**) Hepatic fibrosis in the four groups as detected by Masson’s trichrome (original magnification, × 200). (**C**) Representative images of TUNEL-stained in liver tissues of the four groups (original magnification, × 200). Scale bar = 20 μm. (**D**) Extent of liver injury in the four groups as graded using the Suzuki score. (**E**) Percentage of TUNEL-positive cells in the four groups. *N* = 3, the data are presented as the mean ± SD. *P < 0.05; SRT, SRT1720; sir, sirtinol

The authors apologize for this error and state that this does not change the scientific conclusions of the article in any way.
